# Editorial: Extremism in games

**DOI:** 10.3389/fpsyg.2025.1758296

**Published:** 2026-01-06

**Authors:** Rachel Kowert, Suraj Lakhani, Constance Steinkuehler

**Affiliations:** 1 Department of Psychiatry, University of Cambridge, Cambridge, United Kingdom; 2 School of Law, Politics, and Sociology, University of Sussex, Brighton, United Kingdom; 3 Donald Bren School of Information and Computer Sciences, University of California, Irvine, Irvine, CA, United States

**Keywords:** extremism, games, gaming, misogyny, video games

Over the past decade, the intersection of digital gaming and extremism has emerged as a critical area of concern for researchers, policymakers, technology companies, and international organizations. What was once dismissed as a fringe issue has evolved into a pressing challenge as extremist actors increasingly exploit gaming platforms, gaming-adjacent spaces, and gaming culture for propaganda dissemination, radicalization, recruitment, attack planning, fundraising, and community building.

The goal of this Research Topic was to bring together the latest research examining the intersection of games and extremism. As gaming platforms continue to grow, the urgency of understanding how extremist actors exploit these spaces has intensified. This Research Topic represents one of the first curated assemblies of empirical research specifically focused on extremism in gaming contexts, where alongside deepening existing knowledge, it provides crucial evidence to inform counter-terrorism efforts, platform governance, and prevention strategies.

The articles in this Research Topic align across four broad themes: prevalence and nature of extremist content in gaming spaces, platform governance and moderation challenges, building resilience among gaming communities, and youth vulnerability. Each of these themes is discussed below.

## Prevalence and nature of extremist content

Three articles in this Research Topic provide systematic assessments of extremist content within gaming environments, addressing a critical gap in our empirical knowledge. Kowert et al. conducted a large-scale survey of game players to assess their experiences with extremist sentiment and found that more than half of all respondents reported being either a direct target of or witness to extremist rhetoric within gaming spaces. Extremist content was found to manifest primarily through text-based communication and occurred most frequently in-game, though players also encountered extremism through iconography, images, and voice chat. The findings document exposure to multiple forms of extremism including white supremacy, anti-government sentiment, antisemitism, Islamophobia, misogyny, and LGBTQIA+ agitation.

Miller-Idriss takes a deep dive into the role of misogyny in gaming spaces and online gaming communities “*incubate, channel, and champion*” sexist and misogynist attitudes which has contributed to a mainstreaming of male supremacist ideology and violence. She presents case examples to demonstrate how the mainstreaming of misogyny in these spaces, which are frequented by boys in men, normalizes and mobilizes extremist motivated violence.

Schlegel et al. expanded our understanding by examining a previously understudied area—mod forums. Through an exploratory analysis of 500 posts on Mod DB, one of the most popular modding platforms, the researchers identified hateful and extremist content across multiple ideological strands including right-wing extremism, antisemitism, and Islamism. This work highlights that extremist exploitation of gaming spaces extends beyond mainstream platforms into creative gaming communities, where hateful mods can potentially reach tens of thousands of users.

## Platform governance and moderation challenges

Understanding how platforms respond to extremist content is crucial for developing effective mitigation strategies. Allchorn and Orofino conducted semi-structured interviews with leading practitioners, academics, and content moderation experts to explore how communities are responding to extremist activity on gaming-adjacent platforms such as Discord, Twitch, and Steam. The research reveals that while third-party communities have adopted increasingly sophisticated tactics, their efforts are undermined by the networked and adaptive nature of extremism and by insufficient enforcement mechanisms at the platform level. A particularly concerning finding is the prevalence of “awful but lawful” content, extremist-adjacent material that, while harmful, does not clearly violate platform terms of service. The study calls for greater transparency in content moderation and more robust efforts to counter toxic gaming cultures that create fertile ground for extremist exploitation.

## Building resilience

Given the challenges of completely eradicating harmful content from vast online gaming communities, building individual and community resilience against extremist messaging is essential. Lamphere-Englund et al. developed and tested a novel framework called Building Resilience Against Violent Extremism Digitally (BRAVED), adapting an offline-focused resilience model for digital gaming environments. Drawing on survey data from more than 2,000 gamers across seven countries (Australia, Canada, France, Germany, Indonesia, the UK, and the US), the research identified key factors that contribute to resilience against violent extremism in gaming contexts. Notably, the study employed a gender-based lens, recognizing that male and female gamers may face different vulnerability factors and pathways to extremist content. This work provides actionable insights for developing targeted prevention programs within gaming communities.

## Youth vulnerability

The vulnerability of young people to extremist content in gaming spaces deserves particular attention, given that gaming is especially popular among children and adolescents. Two papers in this Research Topic explored this topic.

Wells et al. conducted a survey among adolescent and young-adult players (13–25 year old) and found that most (85%) reported encountering hate speech online and had witnessed hate-based harassment (82%). Gender-based (69.9%) and ethnicity-based (62.1%) harassment were the most commonly witnessed forms. A key takeaway from this work is that exposure to this kind of material matters. Greater exposure to hate speech significantly increased both productive responses (e.g., supporting victims) and harmful ones (e.g., toxicity in return), and was associated with higher odds of hate-based harassment perpetration. Players from marginalized groups were disproportionately targeted and more likely to withdraw from play, leaving less-affected players behind, conditions that may facilitate the gradual normalization of hate in online gaming spaces.

Hutchinson et al. conducted interviews with experts from government, academic, and education-based organizations across multiple regions to assess professional perspectives on childhood and adolescent exposure to extremist materials in online gaming environments. The research examines how gaming technologies facilitate youth exposure to both extremist content and extremist recruiters, highlighting developmental considerations that make younger users particularly susceptible. The findings underscore the need for age-appropriate educational interventions and stronger protections for minor users on gaming platforms.

## Concluding thoughts and future directions

The work compiled in this Research Topic confirms that extremist rhetoric and behavior have become commonplace in gaming spaces. That said, it is crucial to emphasize that these findings do not suggest that gaming causes radicalization or that games themselves are inherently problematic. Rather, extremist actors are exploiting the massive reach, immersive nature, and social infrastructure of gaming platforms to undertake activities and spread their ideologies.

Gaming represents one of the most significant cultural and social phenomena of our time. For the vast majority of users, gaming provides entertainment, social connection, community, and even mental health benefits. The presence of extremist actors in these spaces should not lead to moral panic or stigmatization of gaming communities. Instead, the research in this Research Topic points toward more nuanced and evidence-based approaches.

Several key themes emerge across these articles that warrant continued attention. First, the ubiquity of extremist content exposure suggests that moderation strategies focused solely on the most egregious violations are insufficient. The normalization of hateful rhetoric, even when it falls short of explicit extremist messaging, may be laying groundwork for more serious radicalization pathways. Second, gaming-adjacent platforms such as Discord and Twitch, are also being used as infrastructure for extremist community building. Third, technical solutions alone will not solve this challenge. Building community resilience, fostering positive gaming cultures, and educating users (particularly young people) about extremist tactics are equally important components of comprehensive prevention strategies.

Looking ahead, there are several research priorities worth of attention. We need more cross-cultural research that examines how gaming and extremism intersect differently across global contexts, particularly in regions beyond North America and Europe. We need closer collaboration between researchers, technology companies, gaming studios, and prevention practitioners to develop evidence-based interventions that can be implemented at scale. We also need continued empirical investigation of emerging trends, including the use of artificial intelligence in content moderation, the role of influencers and streamers in either amplifying or countering extremist narratives, and the exploitation of virtual and augmented reality gaming environments.

Taken together, this Research Topic represents an important step toward building a rigorous empirical foundation for understanding extremism in gaming contexts. By bringing together diverse methodological approaches, from large-scale surveys to in-depth interviews to platform analyses, the complexity of the challenge and the necessity of multi-faceted responses is evident. We hope that the evidence presented here will inform more effective policies, platform governance strategies, and prevention programs, while also inspiring continued research in this growing area of concern.

